# Development of a treatment planning protocol for prostate treatments using intensity modulated radiotherapy

**DOI:** 10.1120/jacmp.v2i2.2614

**Published:** 2001-03-01

**Authors:** Gary A. Ezzell, Steven E. Schild, William W. Wong

**Affiliations:** ^1^ Department of Radiation Oncology Mayo Clinic Scottsdale Scottsdale Arizona 85259

**Keywords:** IMRT, conformal radiotherapy, prostate cancer, treatment planning

## Abstract

We have developed a treatment planning protocol for intensity‐modulated radiation therapy of the prostate using commercially available inverse planning software. Treatment plans were developed for ten patients using the Corvus version 3.8 planning system, testing various prescription options, including tissue types, dose volume histogram values for the target and normal structures, beam arrangements, and number of intensity levels. All plans were scaled so that 95% of the clinical target volume received 75.6 Gy; mean doses to the prostate were typically 79 Gy. The reproducibility of the inverse planning algorithm was tested by repeating a set of the plans five times. Plans were deemed acceptable if they satisfied predefined dose constraints for the targets and critical organs. Figures of merit for target coverage, target dose uniformity, and organ sparing were used to rank acceptable plans. Certain systematic behaviors of the optimizer were noted: the high dose regions for both targets and critical organs were 5–10 Gy more than prescribed; reducing bladder and rectum tolerance increased the range of doses within the target; increasing the number of fields incrementally improved plan quality. A set of planning parameters was found that usually satisfied the minimum requirements. Repeating the optimization with different beam order produced similar but slightly different dose distributions, which was sometimes useful for finding acceptable solutions for difficult cases. The standard set of parameters serves as a useful starting point for individualized planning.

PACS number(s): 87.53.–j, 87.90.+y

## INTRODUCTION

The utility of intensity modulated radiation therapy (IMRT) for the treatment of prostate cancer is being studied by a number of institutions. Recent publications have shown that doses may be increased beyond 70 Gy while keeping the dose to sensitive structures at safe levels.^1^ ^–^ ^6^ Early data have shown improvement in the clinical outcome as a result of applying conformal techniques.^7^ ^–^ ^14^

Planning treatments that go beyond well‐established clinical experience require one to consider a number of difficult questions. What target doses are desired? How much heterogeneity of the target dose can be accepted? What dose‐volume limits for sensitive tissues need to be respected? What margins need to be applied? How can alternative plans be meaningfully compared? This is true for all treatment planning, but IMRT makes the situation even more challenging. The complexity of IMRT requires that sophisticated optimization algorithms be applied in a process termed inverse treatment planning: the planner describes the desired goals and the computer system finds a solution. The planning system may offer additional variables for consideration, such as tissue types, beamlet size, and number of intensity levels, that compound the difficulty of determining the plan parameters. The results of the computation may well not satisfy all the constraints, and there may be clinical considerations that cannot be easily represented by the planning model. Planners must therefore expend considerable effort learning how to use the planning system to best advantage.

This paper describes our experience in developing a protocol to escalate doses for prostate cancer treatments. We first provide the rationale for our choice of specific dose‐volume limits for target and sensitive tissues that delimit acceptable plans, and then we describe criteria for ranking competing plans. We then explain how the abundance of planning variables was progressively winnowed down to establish an efficient, robust planning protocol for these patients.

## MATERIALS AND METHODS

IMRT treatments at the Mayo Clinic, Scottsdale, AZ, are planned using the Corvus system (Sewickley, PA) and delivered with a Varian 2100C (Palo Alto, CA) linear accelerator equipped with a multileaf collimator with 10‐mm‐wide leaves. This study was performed with a prerelease version of updated Corvus software, version 3.8. Plans were run for ten patients who had presented with prostate cancer. The patients were scanned and treated in the supine position. Table I shows the organ volumes for the ten patients studied. The rectum and bladder contours included the entire wall and contents of the lumen. All the plans were calculated using 10‐MV beams and a beamlet size of $10\times 10{\text{mm}}^{2}$. Except where noted, 10% intensity levels were used in the optimization.^15^

**Table I:** Organ volumes (in cm3).

Patient	Prostate	Seminal Vesicles	Rectum	Bladder
*A*	84	33	114	118
*B*	72	19	69	155
*C*	91	38	118	183
*D*	60	36	144	210
*E*	90	13	77	151
*F*	69	22	73	139
*G*	34	5	36	66
*H*	84	20	183	143
*I*	90	13	99	79
*J*	120	18	55	129

Corvus expands the volumes drawn as the clinical target volume (CTV) to form the planning target volume (PTV) using uncertainty dimensions that are specified by the user. Normal structures may be similarly expanded if desired. Geometric uncertainties for immobilization and localization (e.g., organ motion) are combined in quadrature to form the total uncertainty, which may be individually specified in the three principal directions. A search of the relevant literature established the uncertainties used in this protocol. Antolak *et al*.^16^ presented their data along with a review of previous studies. Combining that review with other contemporaneous studies^17^ ^,^ ^26^ ^,^ ^27^ suggests that the uncertainty of localizing the bony anatomy has a standard deviation of 4–5 mm in the anterior‐posterior and superior‐inferior directions and that the uncertainty caused by prostate motion is approximately 3.5 mm in those directions. The published uncertainties are less in the lateral directions (right‐left). The doses to the bladder and rectum are relatively insensitive to expansion of the target in the lateral directions and are most sensitive to the margin applied in the anterior‐posterior direction. Per Antolak et al.,^16^ a 95% probability of covering the CTV can be obtained by setting a planning margin 1.65 times the standard deviation. For simplicity and consistency with previous practice, it was decided to use a uniform uncertainty in each direction of 8 mm for immobilization and 6 mm for localization of the target, resulting in a uniform 10‐mm expansion of the PTV around the CTV. This scheme is generally consistent with the margins quoted by the groups at Memorial Sloan‐Kettering,^1^ Fox Chase,^17^ and 3DOG/RTOG 9406,^12^ although Memorial and Fox Chase reduced the posterior margin at the rectal interface for all or part of the treatment.

Based on published protocols and outcomes,^7^ ^,^ ^8^ ^,^ ^10^ the target dose was set to 75.6 Gy to the prostate for a PSA of less than 10 ng/ml and 77.4 Gy for ≥10 mg/ml using 1.8 Gy/day fractions. For this planning study, the 75.6 Gy target dose was used and the seminal vesicles were prescribed the same dose as the prostate.

Based on reports in the literature concerning rectal,^19^ ^–^ ^22^ bladder,^12^ ^,^ ^20^ ^,^ ^23^ and femoral head^24^ tolerances, the following dose volume limits were established. For the rectum, 40% of the rectal volume could receive ≥65 Gy,30%≥70 Gy, and 10%≥75 Gy. The rectum was contoured from 1.5 cm inferior to the apex of the prostate to the level of the sigmoid at which it changes direction from cephalo‐caudad to anterior‐ posterior. For the bladder, 30% of the volume could receive ≥70 Gy. For both the rectum and bladder, the hotspots could not exceed 81 Gy. Deciding how to define a “hotspot” is difficult. Constraining the highest dose computed to a single voxel may be unduly restrictive. ICRU 50 (Ref. 18) considers a hotspot to be significant if the minimum diameter exceeds 1.5 cm. With that guidance, the hotspot dose was taken to be that given to $1.8{\text{cm}}^{3}$, which is the volume of a 1.5‐cm diameter sphere, recognizing that since the dose volume histogram (DVH) data coalesce all volumes, this high dose volume may not be contiguous. (In the discussions below, maximum to a target or organ refers to this $1.8-{\text{cm}}^{3}$ definition). Finally, the full thickness of the femur could not receive more than 50 Gy. For comparison, the limits used by Reinstein *et al* ^2^ for a target dose of 81 Gy delivered with IMRT were 30% of the rectal wall to 75.6 Gy and 10% to 80 Gy, and 15% of the bladder wall to 80 Gy.

For this planning study, the doses in each plan were scaled so that 95% of the CTV received the target dose. This was deemed preferable to a baseline determined by the PTV because of the overlap between it and the rectum. Since the fraction of PTV overlapping the rectum varies with each patient, one cannot determine a *priori* a minimum fraction to be covered. Also, since the CTV is frequently in contact with the rectum, some compromise in target coverage may be needed to spare the anterior rectal wall.

With this point on the DVH of the CTV held constant, plans were considered acceptable if they met the dose‐volume limits for the normal tissues. In addition, the hotspot dose in the volume could not exceed 115% of the target dose. To further distinguish between acceptable plans, other figures of merit were examined. These included doses of up to 10% and 30% of the rectum, a dose up to 30% of the bladder, and a dose up to 99% of the CTV. (Note that the reported minimum dose to the CTV was not used since a difference in how Corvus interpolates dose and contour boundaries can cause unrealistically low minimum doses to be reported. In the discussion below, a minimum dose to the CTV refers to that which covers 99% of the volume.) We also computed the ratio between the PTV volume and total volume receiving 95% of the target dose. This volume ratio serves as a relative indicator, with higher numbers showing less volume taken to high doses.

## PLANNING RESULTS

Dose prescription in Corvus requires assigning a “tissue type” for each structure along with three points on its cumulative DVH. These three points define the desired maximum dose in the volume, minimum dose in the volume, and one intermediate dose‐volume point defined by a limit dose and the percent of the volume that may be above (for limiting organs) or below (for targets) the limit. Preliminary plans showed that the calculated DVHs systematically had higher maximums than prescribed; the maximum dose actually reported in the volume tended to be about 5 Gy more than requested for either the PTV or the nontarget tissue. For a target dose of 75.6 Gy, the maximum optimization limits for target and normal tissues were therefore set to 81 Gy in order to keep the maximum dose below 115% of the target dose (86.9 Gy). The prescription allowed a minimum of 72 Gy to up to 5% of the target volume.

**Figure 1:**
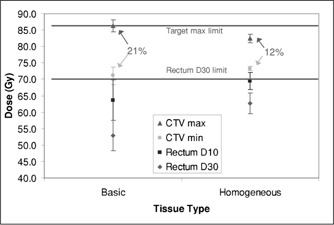
Target and rectal dose parameters for the two target tissue types available.

Plans were then done for the ten patients to test the effects of the various prescription elements sequentially. For a given set of bladder and rectum constraints and a seven field arrangement, plans were completed using the two target tissue types available in Corvus v3.8: “basic” and “homogeneous.” Figure 1 shows that the basic type provided more rectal sparing at the cost of larger dose variation in the target, often exceeding the allowable maximum dose. The homogeneous type spared the rectum less but sufficiently to meet the plan requirements, so the homogeneous target type was used for subsequent planning. (In all these figures, the error bars represent one standard deviation. “D30” refers to the dose given to at least 30% of the organ.)

**Figure 2:**
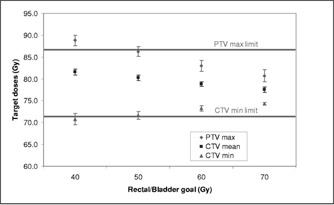
Target dose parameters as a function of organ goals set in prescription.

**Figure 3:**
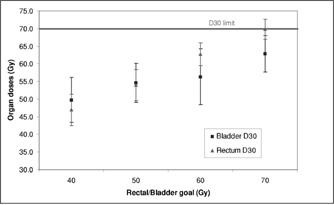
Rectum and bladder dose parameters as a function of organ goals set in prescription.

The next set of plans tested a range of DVH limits for the rectum and bladder using a fixed beam arrangement of seven fields (see Table II). The limit dose to the rectum and bladder varied from 40–70 Gy. The maximum rectum dose was held at 75 Gy, since the rectum is in contact with the target that was to receive 75.6 Gy, and the minimum was set to 30 Gy. For each plan, the prescription allowed 10% of the organ to receive more than the desired limit but less than the maximum value. These plans were evaluated to determine how much the organ dose could be restricted without compromising other treatment goals. Figures 2 and 3 show the effect of these changing limits on target and organ doses. Again, there is a tradeoff between organ sparing and target dose uniformity. Setting the rectum and bladder limit to 60 Gy kept the target and organ doses with the desired limits. With those parameters, the resulting mean dose to the prostate CTV was 78.8±0.5 Gy.

**Table II:** Beam arrangements used.

Plan ID	# beams	Angles (IEC convention)
5 lat	5	65, 90, 180, 270, 295
6 lat	6	40, 90, 115, 245, 270, 320
3 even	3	60, 180, 300
5 even	5	36, 108, 180, 252, 324
7 even	7	25, 75, 130, 180, 230, 285, 335
9 even	9	0, 40, 80, …, (40° intervals)
15 even	15	0, 24, 48, …, (24° intervals)

**Figure 4:**
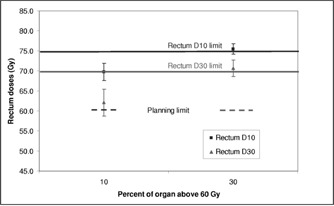
Rectum dose parameters as a function of percentage allowed above 60 Gy in the prescription.

Figure 4 shows the effect of altering the percent of the organ allowed to go above the limit of 60 Gy. The actual rectal dose tends to be about 10 Gy more than that set in the prescription. The 10% prescription level meets the clinical requirements while the 30% level does not.

After the tissue type and prescription DVH values were determined, plans on these ten patients were run to test the relative merit of different beam arrangements. One class of plans used an odd number of beams distributed at equal angular intervals around the axial plane; plans with 3, 5, 7, 9, and 15 beams were tried. The other class used parallel‐opposed laterals with three or four additional beams at selected angles, for a total of 5 or 6 beams. Only axial beams were considered in this study. Table II lists the seven beam arrangements tested.

**Figure 5:**
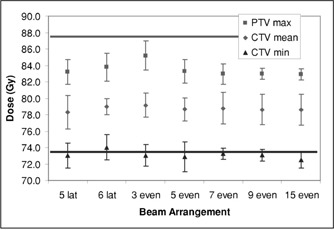
Target dose parameters as a function of beam arrangement.

**Figure 6:**
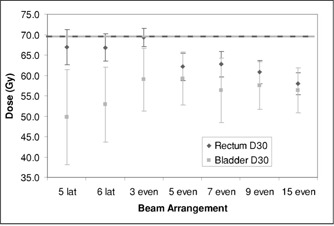
Dose to 30% of the rectum and bladder as a function of beam arrangement.

**Figure 7:**
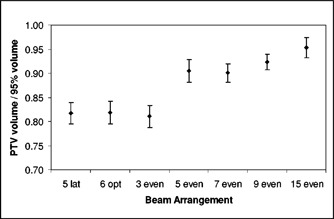
Change in the volume ratio as a function of beam arrangement.

Figure 5 shows that the target dose parameters were relatively unaffected by the beam arrangement. Figure 6 shows that the rectum is better spared by having at least five beams equally distributed in angle, with incremental improvements as the number of fields increases to 9 or 15. Figure 7 shows similar results for the volume ratio; the amount of tissue taken to high dose diminishes with increasing number of fields, with a distinct improvement between three and five fields and slower improvement thereafter.

**Figure 8:**
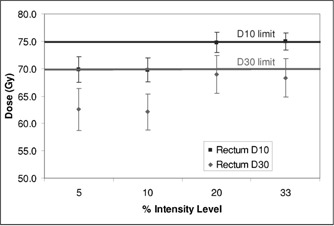
Rectal dose parameters as a function of the size of the modulation step used for the optimization.

Figure 8 shows the results for the “5 even” field arrangement optimized using 5%, 10%, 20%, and 33% intensity levels available for the modulation, which runs from 0–100%. The other prescription parameters were as indicated above. Plan quality for the 5% and 10% levels are similar to each other and better than that for the 20% and 33% levels (p<0.001). The CTV maximum, minimum, and mean doses were very similar and equivalent statistically.

**Figure 9:**
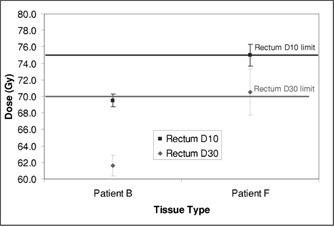
Rectal dose parameters for two patients with plans repeated five times, changing the beam order for each run.

**Figure 10:**
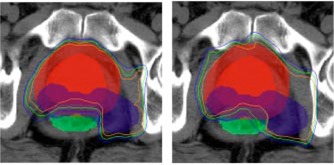
(Color) Isodose lines for two runs of the plan for patient F. The prostate and its PTV are in red, the seminal vesicles are in blue, and the rectum is green. The isodoses lines are for 65, 70, and 75 Gy. The plan in the left panel met the dose criteria; that in the right did not.

Corvus uses a stochastic optimization algorithm based on an implementation of simulated annealing. The results for a plan depend on the random path taken through solution space. Repetitions of the same plan produce virtually identical results because the same random number sequence is used, but the user can force a different path to be followed by permuting the order of beams presented to the optimizer, i.e., renumbering the beams. Plans for the “5 even” beam arrangement were repeated five times for two patients to determine the variability of results for a fixed set of parameters. Patient *B*'s anatomy easily permitted the rectum to be spared while patient *F* had large seminal vesicles that partially wrapped around the anterior rectal wall. As seen in Fig. 9, all of the plans for patient *B* satisfied the rectal dose constraints, while some of Patient *F*'s did and others did not. Figure 10 shows one CT image from two of patient *F*'s plans and the difference in the isodose shapes over the rectum.

The results presented here have concentrated on the rectal doses. Those for the bladder are similar, but there is more variation because of the diversity in shape of bladder and seminal vesicles from patient to patient. Femoral head doses were well within the 50 Gy limit for all patients and plans. Table III summarizes the parameters of the planning protocol.

**Table III:** Parameters of standard planning protocol (Corvus v3.8).

Dose volume limits					
Target/Structure	Tissue type	Goal dose (Gy)	Percent below or above goal	Minimum (Gy)	Maximum (Gy)
Prostate	Homogeneous	75.6	5	72	81
Seminal vesicles	Homogeneous	75.6	5	72	81
Tissue	Homogeneous	75.6	0	0	81
Bladder	Basic	60	10	30	75
Rectum	Basic	60	10	30	75
Femoral heads	Basic	40	10	30	50
Other parameters					
Margins					
Immobilization	8 mm uniform				
Localization	6 mm uniform				
Beam directions	36°, 108°, 180°, 252°, 324° (IEC convention)				
Intensity levels	0–100% in 10% steps				
Beam energy	10 MV				
Beamlet size	$10\times 10{\text{mm}}^{2}$				

## DISCUSSION

Inverse treatment planning is not necessarily a simple matter of prescribing a desired dose distribution. One must learn how to adjust the prescription so that the resulting plan is satisfactory. For example, in this study the maximum dose in the volume typically exceeded the prescribed value by about 5 Gy, so one had to specify a lower maximum than was actually acceptable. One could not overly constrain the rectal dose without causing unacceptable hotspots elsewhere in the volume, as seen in Fig. 2. Reducing the rectal dose means reducing the intensities of some beamlets irradiating the rectum. Where those shadows traverse the target, other beamlets must have higher intensities to compensate. With a limited number of fields, the balancing of high and low intensity beamlets cannot be exact and the result is less dose homogeneity.

For prostate treatments at these dose levels, it was possible to develop a prescription protocol that usually produced plans meeting the minimum requirements that had been established for target and critical organ doses. This standard protocol employs five axial fields, equally distributed in angle, with rectum and bladder D10 limits set to 60 Gy and target doses of 75.6 Gy to 95% of the prostate CTV, leading to a mean prostate dose of 78.8 Gy. The protocol is not “optimal” in the sense of having been proven to be the best combination of prescription parameters for this group of ten patients or even for any one patient. It does, however, provide the planner with a good starting point that will likely produce an acceptable plan (by these criteria), thus simplifying the practical burden of IMRT planning for the prostate. Each revision of the planning parameters requires about 15 minutes to calculate and evaluate. The planner can also use these results to suggest methods of improving plans. Adding beams, for example, is likely to make incremental improvements. Reducing rectum or bladder limits will reduce those doses with concomitantly more dose variation in the target, which may prove to be acceptable. Repeating a plan several times for a challenging patient (by altering the order in which beams are entered into the system) may find a more satisfactory solution. We emphasize that this “standard protocol” is used as a starting point for individualized planning and that the protocol itself is subject to revision as our experience increases.

A number of other planning options could be investigated. Higher energies could be tested. Only axial beams were considered in this study because of the extra treatment time required to change table angles. Nonaxial beams may prove useful if doses are to be escalated higher, but axial beams proved adequate to achieve our dosing protocol. If the prostate were localized for each day's treatment, as by using ultrasound, smaller margins could be applied.^5^ ^,^ ^17^ ^,^ ^25^ Finally, the characteristics of these IMRT plans should be compared to three‐dimensional conformal plans with similar goals and measures. These latter issues are the subject of ongoing investigations in this institution.

## References

[1] C. Burman , C. S. Chui , G. Kutcher , S. Leibel , M. Zelefsky , T. LoSasso , S. Spirou , Q. Wu , J. Yang , J. Stein , R. Mohan , Z. Fuks , and C. C. Ling , “Planning, delivery, and quality assurance of intensity‐modulated radiotherapy using dynamic multileaf collimator: a strategy for large‐scale implementation for the treatment of carcinoma of the prostate,” Int. J. Radiat. Oncol., Biol., Phys. 39, 863–873 (1997). 9369136 10.1016/s0360-3016(97)00458-6

[2] L. E. Reinstein , X. H. Wang , C. M. Burman , Z. Chen , R. Mohan , G. Kutcher , S. A. Leibel , and Z. Fuks , “A feasibility study of automated inverse treatment planning for cancer of the prostate,” Int. J. Radiat. Oncol., Biol., Phys. 40, 207–214 (1998). 9422578 10.1016/s0360-3016(97)00582-8

[3] B. Pickett , E. Vigneault , J. Kurhanewicz , L. Verhey , and M. Roach , “Static field intensity modulation to treat a dominant intra‐prostatic lesion to 90 Gy compared to seven field 3‐dimensional radiotherapy,” Int. J. Radiat. Oncol., Biol., Phys. 43, 921–929 (1999). 10386651 10.1016/s0360-3016(98)00502-1

[4] B. S. Teh , S. Y. Woo , and E. B. Butler , “Intensity modulated radiation therapy (IMRT): a new promising technology in radiation oncology,” The Oncologist 4, 433–442 (1999). 10631687

[5] M. J. Zelefsky , Z. Fuks , L. Happersett , H. J. Lee , C. C. Ling , C. M. Burman , M. Hunt , E. S. Venkatraman , A. A. Jackson , and S. A. Leibel , “Improved conformality and reduced toxicity with high‐dose intensity modulated radiation therapy (IMRT) for patients with prostate cancer,” Int. J. Radiat. Oncol., Biol., Phys. 45, 170 (1999).

[6] D. S. Mohan , P. A. Kupelian , and T. R. Willoughby , “Short‐course intensity‐modulated radiotherapy for localized prostate cancer with daily transabdominal ultrasound localization of the prostate gland,” Int. J. Radiat. Oncol., Biol., Phys. 46, 575–580 (2000). 10701736 10.1016/s0360-3016(99)00454-x

[7] A. Pollack and G. K. Zagars , “External beam radiotherapy dose response of prostate cancer,” Int. J. Radiat. Oncol., Biol., Phys. 39, 1011–101 (1997). 9392538 10.1016/s0360-3016(97)00508-7

[8] M. J. Zelefsky , S. A. Leibel , G. J. Kutcher , and Z. Fuks , “Three‐dimensional conformal radiotherapy and dose escalation: Where do we stand?” Semin Radiat. Oncol. 8, 107–114 (1998). 9516591 10.1016/s1053-4296(98)80006-4

[9] G. E. Hanks , A. H. Hanlon , T. E. Schultheiss , W. H. Pinover , B. Movsas , B. E. Epstein , and M. A. Hunt , “Dose escalation with 3D conformal treatment: five year outcomes, treatment optimization, and future directions,” Int. J. Radiat. Oncol., Biol., Phys. 41, 501–510 (1998). 9635695 10.1016/s0360-3016(98)00089-3

[10] G. E. Hanks , A. L. Hanlon , W. H. Pinover , E. M. Horwitz , and T. E. Schultheiss , “Survival advantage for prostate cancer patients treated with high‐dose three‐dimensional conformal radiotherapy,” Cancer J. Sci. Am. 5, 145–6 (1999). 10367171

[11] M. J. Zelefsky , S. A. Leibel , P. B. Gaudin , G. J. Kutcher , N. E. Fleshner , E. S. Venkatramen , V. E. Reuter , W. R. Fair , C. C. Ling , and Z. Fuks , “Dose escalation with three‐dimensional conformal radiation therapy affects the outcome in prostate cancer,” Int. J. Radiat. Oncol., Biol., Phys. 41, 491–500 (1998). 9635694 10.1016/s0360-3016(98)00091-1

[12] J. M. Michalski , J. A. Purdy , K. Winter , M. Roach , S. Vijayakumar , H. M. Sandler , A. M. Markoe , M. A. Ritter , K. J. Russell , S. Sailer , W. B. Harms , C. A. Perez , R. B. Wilder , G. E. Hanks , and J. D. Cox , “Preliminary report of toxicity following 3D radiation therapy for prostate cancer on 3DOG/RTOG 9406,” Int. J. Radiat. Oncol., Biol., Phys. 46, 391–402 (2000). 10661346 10.1016/s0360-3016(99)00443-5

[13] P. A. Kupelian , D. S. Mohan , J. Lyons , E. A. Klein , and C. A. Reddy , “Higher than standard radiation doses (≥72 Gy) with or without androgen deprivation in the treatment of localized prostate cancer,” Int. J. Radiat. Oncol., Biol., Phys. 46, 567–574 (2000). 10701735 10.1016/s0360-3016(99)00455-1

[14] D. P. Dearnaley , V. S. Khoo , A. R. Norman , L. Meyer , A. Nahum , D. Tait , J. Yarnold , and A. Horwich , “Comparison of radiation side‐effects of conformal and conventional radiotherapy in prostate cancer: a randomised trial,” Lancet 353, 267–272 (1999). 9929018 10.1016/S0140-6736(98)05180-0

[15] L. Xing , B. Curran , R. Hill , T. Holmes , L. Ma , K. M. Forster , and A. L. Boyer , “Dosimetric verification of a commercial inverse treatment planning system,” Phys. Med. Biol. 44, 463–478 (1999). 10070795 10.1088/0031-9155/44/2/013

[16] J. A. Antolak , I. I. Rosen , C. H. Childress , G. K. Zagars , and A. Pollack , “Prostate target volume variations during a course of radiotherapy,” Int. J. Radiat. Oncol., Biol., Phys. 42, 661–672 (1998). 9806528 10.1016/s0360-3016(98)00248-x

[17] J. Lattanzi , S. McNeely , A. Hanlon , I. Das , T. E. Schulteiss , and G. E. Hanks , “Daily CT localization for correcting portal errors in the treatment of prostate cancer,” Int. J. Radiat. Oncol., Biol., Phys. 41, 1079–1086 (1998). 9719118 10.1016/s0360-3016(98)00156-4

[18] International Commission on Radiation Units and Measurements , ICRU Report 50: Prescribing, Recording, and Reporting Photon Beam Therapy (Bethesda, Maryland, 1993).

[19] A. C. Hartford , A. Niemierko , J. A. Adams , M. M. Urie , and W. U. Shipley , “Conformal irradiation of the prostate: estimating long‐term rectal bleeding risk using dose‐volume histograms,” Int. J. Radiat. Oncol., Biol., Phys. 36, 721–730 (1996). 8948358 10.1016/s0360-3016(96)00366-5

[20] L. J. Boersma , M. Van den Brink , A. M. Bruce , T. Shouman , L. Gras , A. T. Velde , and J. V. Lebesque , “Estimation of the incidence of late bladder and rectum complications after high‐dose (70–78 Gy) conformal radiotherapy of prostate cancer, using dose‐volume histograms,” Int. J. Radiat. Oncol., Biol., Phys. 41, 83–92 (1998). 9588921 10.1016/s0360-3016(98)00037-6

[21] M. R. Storey , A. Pollack , G. Z. Zagars , L. G. Smith , J. A. Antolak , and I. I. Rosen , “Complications from dose escalation in prostate cancer: preliminary results of a randomized trial,” Int. J. Radiat. Oncol., Biol., Phys. 45, 170–171 (1999). 10.1016/s0360-3016(00)00700-8 11020558

[22] M. W. Skwarchuk , A. Jackson , M. Zelefsky , E. Venkatraman , D. Cowen , C. Burman , Z. Fuks , S. Leibel , and C. Ling , “Correlation of DVH and treatment planning variables with late rectal bleeding after 3D‐CRT of prostate cancer: What is the best way to define the rectal length and volume?” Int. J. Radiat. Oncol., Biol., Phys. 45, 261 (1999).

[23] L. B. Marks , P. R. Carroll , T. C. Dugan , and M. S. Anscher , “The response of the urinary bladder, urethra, and ureter to radiation and chemotherapy,” Int. J. Radiat. Oncol., Biol., Phys. 31, 1257–1280 (1995). 7713787 10.1016/0360-3016(94)00431-J

[24] B. Emami , J. Lyman , A. Brown , L. Coia , M. Goitein , J. E. Munzenrider , B. Shank , L. J. Solin , and M. Wesson , “Tolerance of normal tissue to therapeutic irradiation,” Int. J. Radiat. Oncol., Biol., Phys. 21, 109–122 (1991). 2032882 10.1016/0360-3016(91)90171-y

[25] J. Lattanzi , S. McNeeley , A. Hanlon , T. E. Schultheiss , and G. E. Hanks , “Ultrasound‐based stereotactic guidance of precision conformal external beam radiation therapy in clinically localized prostate cancer,” Urology 55, 73–78 (2000). 10654898 10.1016/s0090-4295(99)00389-1

[26] A. Tinger , J. M. Michalski , A. Cheng , D. A. Low , R. Zhu , W. R. Bosch , J. A. Purdy , and C. A. Perez , “A critical evaluation of the planning target volume for 3‐D conformal radiotherapy of prostate cancer,” Int. J. Radiat. Oncol., Biol., Phys. 42, 213–221 (1998). 9747840 10.1016/s0360-3016(98)00189-8

[27] L. A. Dawson , K. Mah , E. Franssen , and G. Morton , “Target position variability throughout prostate radiotherapy,” Int. J. Radiat. Oncol., Biol., Phys. 42, 1155–1161 (1998). 9869243 10.1016/s0360-3016(98)00265-x

